# New Perspectives in the Association between Anthropometry and Mortality: The Role of Calf Circumference

**DOI:** 10.14283/jfa.2024.4

**Published:** 2024-01-24

**Authors:** Chiara Ceolin, V. Acunto, C. Simonato, S. Cazzavillan, M. Vergadoro, M.V. Papa, G.S. Trapella, R. Sermasi, M. Noale, M. De Rui, B.M. Zanforlini, C. Curreri, A. Bertocco, M. Devita, A. Coin, G. Sergi

**Affiliations:** 1 Division of Geriatrics, Department of Medicine (DIMED), University of Padua, Via Giustiniani 2, 35128, Padua, Italy; 2 School of Community Medicine and Primary Care, Department of Women's and Children's Health, University of Padua, Padua, Italy; 3 Neuroscience Institute, National Research Council, Padua, Italy; 4 Department of General Psychology (DPG), University of Padua, Padua, Italy

**Keywords:** Older adults, mortality, calf circumference, sarcopenia, hospitalization

## Abstract

**Aims:**

Considering the impact of sarcopenia on mortality, and the difficulty to assessment of body composition, the hypothesis of the study is that calf circumference (CC) is closely related to mortality in older patients. The aim of the study was to analyze the potential role of CC to predict mortality in old individuals at 3, 6 and 12 months after discharge from hospital.

**Methods:**

Patients aged >65 years were recruited for this retrospective study from September 2021 to March 2022. Their physical and body composition characteristics (including Body Mass Index-BMI and Mini Nutritional Assessment-MNA) were measured; data on mortality at 3 (T3), 6 (T6) and 12 (T12) months after discharge were recorded. Sarcopenia was diagnosed according to the 2019 European Consensus criteria.

**Results:**

Participants were 192 older adults (92 women), with a mean age of 82.8±7.0 years. Sarcopenic people were 41. The mortality rate was higher in sarcopenic people only at T3 and T6. CC had comparable validity in predicting mortality to that of MNA and ASMMI (Appendicular Skeletal Muscle Mass), and was better than BMI and serum albumin at each time point. Youden's index showed that the best cut-off for CC for predicting mortality was 30.6 cm both at T3 (sensitivity: 74%; specificity: 75%) and T6 (sensitivity: 75%; specificity: 67%). At the Cox regression model for mortality, high values of CC (HR 0.73, CI95% 0.60–0.89/p<0.001) and ADL scores (HR 0.72, CI95% 0.54–0.96/p=0.04) were protective factors at T6 and T12 respectively; at T12 high comorbidity rate was a risk factor (HR 1.28, IC95% 1.02–1.62/p=0.04).

**Conclusions:**

CC has a validity comparable to MNA and ASMMI in predicting mortality at 3, 6 and 12 months after hospital discharge. Moreover, it can be considered an independent predictor of medium-term mortality in the hospitalized older population. CC can be an effective method for the prognostic stratification of these patients, due to its simplicity and immediacy.

## Introduction

**T**he term sarcopenia refers to the age-related structural and functional decline of skeletal muscle (1). Sarcopenia is a recognized independent risk factor for numerous poor outcomes: although the pathophysiological mechanisms underlying sarcopenia are not fully understood, the relapse of inflammatory cytokines in sarcopenic people seems to be involved, which in turn leads to a cascade of adverse events, such as bed rest prolongation and infections (2). Moreover, sarcopenia reduces functional performance with impact on quality of life (3), falls, trauma and fractures, and it is associated to frequent and long hospitalizations, and high morbidity burden and mortality (4).

In recent decades attempts have been made to correlate sarcopenia with other parameters, such as anthropometric indices, and calf circumference (CC) has emerged as the one with the strongest associations (5, 6, 7). Together with arm circumference, CC reflects subcutaneous fat and bone mass (8), but is considered a particularly good indicator of body muscle mass: this is because the legs contain over half the muscle mass of the body, which is frequently and directly compromised by reduced walking during illness (8, 9, 10). This makes CC a surrogate marker of muscle mass for diagnosing sarcopenia as well (5, 11). Santos et al. (12) evaluated about 15,000 adults in community settings and found relatively high correlations between CC and appendicular skeletal muscle mass (ASMM) measured by dual-energy X-ray absorptiometry. Similarly, Gonzales-Correa et al. found significant moderate positive correlations between CC and ASMM measured by bioelectrical impedance analysis BIA (13). The results were confirmed in hospital settings (7, 14).

Given the relationships between CC and sarcopenia, and between sarcopenia and mortality, we would expect CC to play a role in predicting mortality. Previously, different studies reported the association between CC and mortality in older adults, also proposing heterogeneous cut-off values that defined the risk profile (15, 16, 17). To our knowledge, the Italian studies about the role of CC in predicting mortality are lacking, and no one directly compared the validity of this anthropometric tool with other indices currently in use for estimating a state of malnutrition, i.e. Mini Nutritional Assessment (MNA), Body Mass Index (BMI) or serum albumin.

Given these premises, the hypothesis of the study is that CC may be closely related to mortality in the older adults and that its validity in predicting mortality may be similar or better to that other malnutrition indices. Therefore, the primary aim of the study was to compare to MNA, BMI and serum albumin the potential of CC to predict mortality in older individuals hospitalized for any cause at 3, 6 and 12 months after discharge; secondarily, we aimed to estimate the most accurate value to adopt as a risk cut-off.

## Materials and Methods

### Study Population

This retrospective study was conducted with a consecutive series of Caucasian patients recruited at the UOC Geriatria of the Azienda Ospedale Università Padova from September 2021 to March 2022. Participants were patients over 65 years of age, regardless of their admission diagnosis. The only inclusion criterion was that we could evaluate their body composition using bioelectrical impedance analysis; exclusion criteria were: fever status, severe dehydration, heart failure with important body edema, patients with unstable medical conditions (e.g., uncontrolled cardiac arrhythmias, uncontrolled hypertension, recent myocardial infarction or angina pectoris, hemodynamic instabilities, or severe dementia) or those who had been hospitalized within three months before the assessment.

The study protocol was conducted in accordance with good clinical practice guidelines and the ethical standards of the 1964 Declaration of Helsinki as revised in 2000, and was approved by the local Ethics Committee (Comitato Etico per la Sperimentazione Clinica della Provincia di Padova, protocol number 16412/AO/23). The subjects taking part in the study were given a detailed explanation of the risks and benefits of participation, and all gave oral and written informed consent to publication of the data.

### Data collection

The following information was collected from each participant by trained physicians.

#### Patient characteristics

Physiological, clinical and pharmacological data were collected during a medical interview by skilled physicians. These included smoking and alcohol habits, social and environmental conditions, and symptoms and diagnosis at admission; comorbidities were assessed with the Cumulative Illness Rating Scale (CIRS) (18). In addition, the patients' functional autonomy was assessed with the Activities of Daily Living (ADL) (19) and the Instrumental Activities of Daily Living (IADL) (20), nutritional status with the MNA (21), and cognitive performance with the Mini Mental State Examination (MMSE) (22). Information on mortality was collected at 3 (T3), 6 (T6) and 12 (T12) months after discharge.

#### Anthropometry

All measures were recorded at the admission of the patients, within the first 24–24 hours, in order to exclude the worsened physical function due to hospitalization. Body weight was measured to the nearest 0.1 kg, using a standard balance with individuals wearing light clothes and no shoes; for those unable to walk, a lift scale was used. As most people were unable to maintain an upright position, body height was calculated from knee-to-heel length according to the Chumlea's equations (23). BMI was calculated as the ratio between weight (kg) and height squared (meters). CC was measured at the maximum circumference of the dominant calf (24), keeping the individuals in a supine position with the knee bent at 90°, using a measuring tape at the point of greatest diameter. An experienced physician checked for pitting edema before CC measurement; furthermore, the measurements were all obtained in the morning in order to reduce the effect of edema. The feet were placed on the bed with the feet and ankles relaxed. Mid-upper arm circumference (MUAC) was measured on the dominant upper arm at the midpoint between the tip of the shoulder and the tip of the olecranon process (25).

#### Muscle strength measurement

Upper limb strength was evaluated with DynEx electronic hand dynamometers (Ohio, USA) by trained medical personnel. Three tests were carried out for each hand, and grip strength was calculated as the mean of the maximum performance at the dominant and non-dominant hand.

#### Evaluation of body composition

Whole-body tetrapolar bioelectrical impedance analysis (BIA) was performed using a BIA 101 Anniversary analyzer (AKERN/RJL Systems, Florence, Italy) with an alternating sinusoidal electric current of 400 µA at a single operating frequency of 50 kHz. The device was calibrated every morning using the standard control circuit supplied by the manufacturer with a known impedance (resistance = 380 Ω; reactance = 47 Ω). The device had a precision of 1% for resistance (Rz), and 5% for reactance (Xc). Participants kept resting in the supine position for at least 5–10 minutes prior to the examinations. The BIA was performed with subjects supine and their limbs slightly away from their body, after an overnight fast and bladder voiding. To avoid inter-observer errors, all BIA measurements were taken by the same trained investigator. Active electrodes (BIATRODES®, Akern Srl, Florence, Italy) were placed on the right side on conventional metacarpal and metatarsal lines, and recording electrodes in standard positions on the right wrist and ankle (26). All resistance measurements were normalized for stature (height in centimeters squared/Rz) to obtain the resistive index (RI). The repeatability and accuracy of the resistance and reactance measurements allowed the smallest changes to be recorded at a resolution of 0.1 Ω. Fat-free mass (FFM) and appendicular skeletal muscle mass (ASMM) estimates for our sample were calculated using the equations developed by Kyle et al. (27, 28), i.e. ASMM = −4.211 + (0.267*RI) + (0.095*weight) + (−0.012*age) + (0.058*reactance) + (1.909*sex), and FFM = −4.104 + (0.518*RI) + (0.231*weight) + (0.130*reactance) + (4.229*sex), where men = 1 and women = 0. Fat mass (FM) was calculated as Total Weight – (FFM+ASMM). The skeletal appendicular muscle mass index (ASMMI) and the fat mass index (FMI) were obtained by dividing the ASMM and FM, respectively, by the subject's height in meters squared.

### Definition of sarcopenia

Sarcopenia was diagnosed on the basis of the muscle strength and mass values according to the criteria of the revised European consensus on definition and diagnosis of 2019 (29). Upper limb strength values below 16 kgf for women and 27 kgf for men at maximum handgrip strength were taken as indicating probable sarcopenia, which was confirmed if the ASMMI values were less than 5.5 kg/h^2^ for women and 7.0 kg/h^2^ for men.

### Statistical analysis

The characteristics of the sample are expressed as means ± standard deviation for the continuous quantitative variables with a normal distribution, and as medians (interquartile range) for the variables with a non-normal distribution. The normality of the distributions of the continuous quantitative variables was assessed by the Shapiro-Wilk test. Categorical variables were expressed as counts and percentages. The characteristics of the study participants were compared with the Student's t-test for independent samples for parametric variables, the Wilcoxon rank sum test test for non-parametric variables, and the Chi-square or Fisher's test for categorical variables.

Receiver operating characteristic (ROC) curve analyses were performed for CC, MNA, BMI, and albumin values. The area under the curve (AUC) and Youden's index were used to determine the values that presented the best balance between sensitivity and specificity for mortality after 3, 6 and 12 months.

To control for the effect of confounding variables in predicting mortality (i.e. gender, age, hospital stay, number of drugs, comorbidities), Cox regression analyses were run, and the effect of each variable was evaluated after adjusting for the other variables. Because of the criteria of recruitment (i.e. patients regardless their diagnosis of admission), we adjusted our Cox model using the CIRS-CI score, including the severity of the entry pathologies. Considering that all patients were taking polytherapy, i.e. more than 5 drugs on average, we considered the continuous variable relating to the absolute number of drugs indicated at discharge. The hazard ratios (HR) and 95% confidence intervals (CI) are also reported. Finally, we performed a sensitivity analysis in order to verify the results obtained, considering the presence of sarcopenia, levels of independence (evaluated by ADL) or disease status (evaluated by CIRS-CI): for this reason, we considered different subpopulations, based on low ADL score (defined as ADL≤3) and more comorbidities (CIRS-CI≥5).

The statistical tests were considered significant at p <0.05. All analyses were performed in IBM SPSS Statistics version 29.0 (IBM Corp., Armonk, NY, USA).

## Results

Table 1 shows the characteristics of our sample at baseline divided by presence of sarcopenia. Sarcopenic people were older and more functionally and cognitively compromised. No difference in albumin levels were recorded between the two groups. Anthropometric parameters, i.e. BMI, calf and arm circumferences, were lower in sarcopenic patients (p<0.001).

**Table 1:** Characteristics of the sample at baseline according to the presence of sarcopenia

Variable	Whole sample (n=192)	Sarcopenic (n=41)	Non sarcopenic (n=151)	p-value
Age [years]	82.8±7.0	85.7±5.6	81.4±7.1	<0.001
Women [%]	92 (47.9%)	15 (36.6%)	77 (51.0%)	0.19
N drugs	6.00 (3.00;8.00)	6.00(4.00;9.00)	6.00(3.00;8.00)	0.29
CIRS-CI	3.00 (2.00;5.00)	3.00 (2.00;5.00)	3.00 (2.00;5.00)	0.27
Hospital stay	16.50 (11.00;25.00)	20.00 (12.00;29.00)	16.00 (12.00;23.00)	0.09
Diagnosis at admission				0.91
Respiratory	39 (20.3%)	10 (24.4%)	29 (19.2%)	
Cardiovascular	22 (11.5%)	6 (14.6%)	16 (10.6%)	
Infections/sepsis	61 (31.8%)	12 (29.3%)	49 (32.5%)	
Abdominal	6 (3.1%)	2 (4.9%)	4 (2.6%)	
Stroke	5 (2.6%)	2 (4.9%)	3 (2%)	
Syncope	14 (7.3%)	2 (4.9%)	12 (8%)	
Other	45 (23.4%)	7 (17.1%)	38 (25.2%)	
Social Context				0.75
Living with family	125 (71.7%)	20 (48.8%)	105 (69.5%)	
Living alone	49 (28.3%)	13 (31.7%)	36 (23.8%)	
Functional evaluation				
Max. handgrip strength [Kgf]	17.80 (11.85;23.35)	15.50 (10.90;19.90)	19.50 (13.60;28.50)	<0.001
IADL	1.00 (0.00;5.00)	1.00 (0.00;2.00)	2.00 (1.00;6.00)	0.01
ADL	1.00 (1.00;5.00)	1.00 (0.00;2.00)	2.00 (1.00;6.00)	0.001
MNA	17.91±4.36	14.96±4.10	18.78±4.12	<0.001
Albumin [g/L]	31.00 (28.00;34.00)	29.50 (28.00;33.00)	31.00 (28.25;34.00)	0.09
MMSE	23.70 (2.00;5.00)	21.30 (14.00;24.15)	25.40 (19.90;27.73)	0.003
Body composition evaluation				
ASMMI	6.65±1.14	5.79±0.87	6.99±1.06	<0.001
BMI [Kg/m^2^]	27.2±6.6	24.9±3.5	30.1±5.8	<0.001
MUAC [cm]	27.00 (24.00;29.00)	25.00 (22.75;26.25)	28.00 (26.00;31.00)	0.001
Calf circumference [cm]	32.1±3.9	29.1±3.1	33.3±3.5	<0.001


Notes: Values are expressed as means ± standard deviation, median (interquartile range) or counts (percentages %) as appropriate. Abbreviations: CIRS-CI = Cumulative Illness Rating Scale - Comorbidity Index; IADL = Instrumental Activities of Daily Living; ADL = Activities of Daily Living; MNA = Mini Nutritional Assessment; MMSE = Mini Mental State Examination; ASMMI = Appendicular Skeletal Muscle Mass Index; BMI = Body Mass Index; MUAC = Mid-Upper Arm Circumference.

The mortality rate was higher in sarcopenic people both at T3 (17.1% vs 4.9%, p=0.03) and T6 (24.4% vs 7.8%, p=0.01). At T12, about 24% of the sample was dead, but no significant differences between sarcopenic and no sarcopenic people were recorded. Regarding gender differences, we observed higher mortality rates in men, but with no statistically significant differences between men and women during the observational period. Between T3 and T6 there was a considerable increase in mortality in women (7.6% vs 14%, p=0.05), while the rates were more than doubled for men between T6 and T12 (11% vs 26%, p=0.05, data not shown).

CC was strongly correlated with both the ASMMI (r=0.603, p=0.001) and maximum handgrip strength (r=0.564, p=0.001), data not shown.

**Figure 1:**
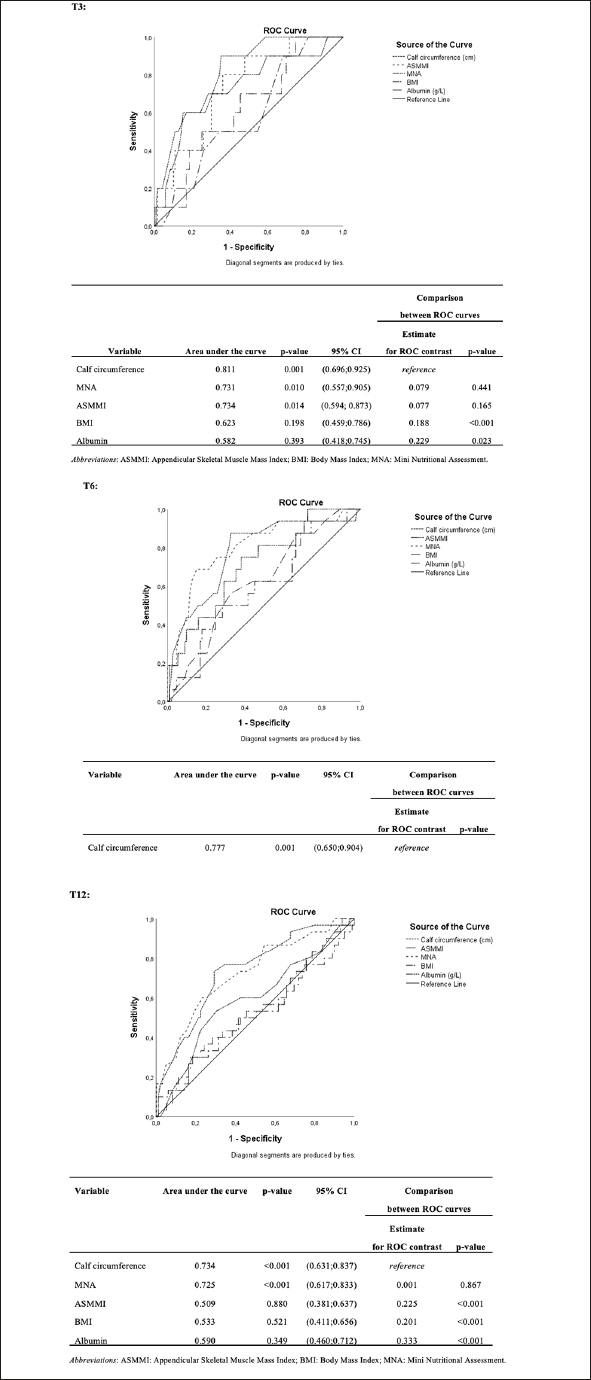
Comparison of accuracy of calf circumference, ASMMI, MNA, albumin, and BMI in predicting mortality at T3, T6 and T12: ROC curves

The diagnostic accuracy of CC, MNA, ASMMI, albumin, and BMI in predicting mortality was assessed by analyzing the respective ROC curves. CC was shown to have comparable validity to ASMMI and MNA and was better than BMI and albumin at each time point; at T12, CC was even superior than ASMMI (Figure 1). Youden's index showed that the best prediction value was a calf diameter of 30.6 cm at T3 (sensitivity: 74%; specificity: 75%) and T6 (sensitivity: 75%; specificity: 67%), while at T12 was 30.7 cm (sensitivity: 73%; specificity: 71%).

The trend in the association between CC and mortality at all the observational time points is shown in Supplementary Figure 1.

Cox regression adjusted for sample characteristics (age, gender, number of drugs at discharge, comorbidities, functional status) revealed that at 6 and at 12 months after discharge significant protective factors were high CC values (Table 2: HR 0.73, 95% CI 0.60–0.89, p<0.001) and high ADL scores (HR 0.72, 95%CI 0.54–0.96, p=0.02); moreover, at T12 high comorbidity rates were risk factors (HR 1.28, 95%CI 1.02–1.62, p=0.04). We considered also the variable on diagnosis at admission both in the univariable and in the multivariable Cox-regression models, and we did not find significant association both at T6 (univariable model, p=0.6231; multivariable model, p=0.1881) and at T12 (univariable model, p=0.7827; multivariable model, p=0.7237, data not shown). Analyzing the AUC and the contrasts between one model containing CC and one model containing ASMMI, we did not find differences that for all time points between the models (data not shown). Sensitivity analyses conducted for different independence status and comorbidity degree confirmed that CC was protective factor at T6 in patients with functional impairment (ADL≤3), while comorbidities did not influence the role of the anthropometric parameter. Finally, a sensitivity analyses was performed also considering the presence of sarcopenia, confirming the protective role of CC at T6 and the influence of ADL scores and comorbidity at T12 (data not shown).

**Table 2:** Cox regression of covariate-adjusted mortality

At T6					
Outcome	Variable	HR	p-value	IC 95%	
				Lower limit	Upper limit
Mortality per 1 point increase in variable	Age [years]	1.00	0.92	0.90	1.11
	N. drugs at discharge	0.95	0.65	0.75	1.20
	CIRS-CI	1.21	0.35	0.82	1.78
	Hospital stay	0.94	0.07	0.88	1.01
	High ADL	0.74	0.18	0.47	1.15
	**Calf circumference [cm]**	0.73	<0.001	0.60	0.89
Mortality per specified category	Gender F	0.57	0.42	0.15	2.22
**At T12**					
**Outcome**	**Variable**	**HR**	**p-value**	**IC 95%**	
				**Lower limit**	**Upper limit**
Mortality per 1 point increase in variable	Age [years]	1.02	0.53	0.96	1.09
	N. drugs at discharge	1.08	0.25	0.95	1.23
	**CIRS-CI**	1.28	0.04	1.02	1.62
	Hospital stay	1.00	0.94	0.97	1.04
	**High ADL**	0.72	0.02	0.54	0.96
	Calf circumference [cm]	0.92	0.16	0.82	1.03
Mortality per specified category	Gender F	0.79	0.59	0.34	1.85


Abbreviations: HR = Hazard Ratio; CIRS-CI = Cumulative Illness Rating Scale - Comorbidity Index; ADL = Activities of Daily Living.

## Discussion

Our study shows that CC, an anthropometric parameter significantly associated with sarcopenia, has a good level of accuracy in predicting mortality 3 and 6 months after hospital discharge. Its validity is comparable to that of MNA, suggesting that CC could be used as a prognostic tool which summarizes data on body composition and nutritional status. Low CC levels are important risk factors for mortality in older adults 6 months after discharge, regardless of the diagnosis of a sarcopenic status. In the long period, other factors influence the outcome of older patients, such as functional independence and comorbidities.

Sarcopenia, i.e. the loss of muscle mass and strength, is the result of the interaction between aging and various risk factors (constitutional, lifestyle-related, and those associated with the patient's burden of morbidity) which contributes to defining sarcopenia as a geriatric syndrome (30). With the demographic aging of the population, therefore, the prevalence of sarcopenia is destined to increase (30). Factors such as reduced physical activity and/or inadequate nutrient intake during hospitalization play an important role in worsening preexisting sarcopenic conditions, leading to conditions such as frailty, disability, and death (31, 32). In line with these findings, patients with sarcopenia in our study were the most functionally and cognitively compromised, but no great differences were detected on comorbidity index and admission diagnosis between sarcopenic and no sarcopenic people, in contrast with other studies (33). The mortality rate was higher in sarcopenic patients especially 3 and 6 months after discharge, as previously reported (32).

According to the most recent guidelines, the best way to assess muscle quality is though instrumental examination, i.e., computed tomography (CT), magnetic resonance imaging (MRI), dual-energy X-ray absorptiometry (DXA), and bioelectrical impedance analysis (BIA) (34). However, considering the difficulty in accessing instrumental resources, especially where the patient is frail and difficult to transport or is uncooperative, it is essential that alternative methods are available to assess body composition (35). Anthropometry allows for fast, low-cost, non-invasive evaluation of the dimensions, proportions and composition of the body using tools that are readily available and easy to transport (34). The World Health Organization (WHO) considers CC to be the most sensitive anthropometric indicator of muscle mass (36) as the lower limbs contain more than half of the body's muscle mass. Furthermore, there are smaller amounts of adipose tissue in the leg: its circumference is almost exclusively influenced by muscle mass, making CC the most representative anthropometric measurement of body muscle mass (37). In line with our results, significant correlations between reduced CC and the two elements that define sarcopenia (i.e. reduced muscle strength and muscle quality) have been observed, including in older populations (15, 38). It may therefore be concluded that CC measurements can be used in the diagnosis of sarcopenia (29).

Given the high impact of sarcopenia on mortality, we hypothesized that CC would also play a role here. Our ROC curves for CC indicated that it played a similar role to MNA, and a bigger role than BMI and albumin in predicting mortality at 3, 6 and 12 months. To our knowledge, no studies have directly compared CC and MNA score indices in predicting mortality, as CC is usually only incorporated into more complex tools and used only in predicting malnutrition (39). For example, Beretta et al reported that the diagnosis of malnutrition through the Global Leadership Initiative on Malnutrition (GLIM) using CC had the best accuracy (AUC = 0.70; 95% CI, 0.63–0.79) and sensitivity (95.8%) to predict in-hospital mortality in older surgical patients than MNA Long Form (40). In our study, a specific circumference of 30.6 cm was identified as the best cut-off value in terms of sensitivity and specificity. Considering a CC value below 31 cm the cut-off usually correlated with malnutrition according to the MNA, our findings may explain the partial overlap in the accuracy of MNA and CC, suggesting that calf measurement plays a role in predicting not only sarcopenia, but also nutritional status. Our results about the superiority of CC than BMI are in line with other studies: in fact, BMI has lower accuracy in older adults due to the changes in body composition these patients undergo over the years (41, 42), and to the difficulty these people may have in standing for height and weight measurements (6). Regarding albumin, a Chinese study reported good discrimination in predicting mortality among immobile patients only for the association between anthropometric and biochemical measures, while CC was more reliable for single measures of accuracy (43). Finally, based on what was previously said, the superiority of CC over ASMMI 12 months after discharge should not be surprising: ASMMI is defined as the sum of the lean muscle mass of the upper and lower extremities adjusted with height (27). In our study, only CC was strictly associated with mortality, while MUAC had no relationship. This might be explained by a “less biased” association with muscle mass than other body measures, because CC is usually less affected by fat deposits (35). It is therefore possible that, as a surrogate for both sarcopenia and malnutrition, the muscle mass of the lower limbs plays a greater role than that of the upper limbs.

In line with previously reports (15, 16, 17, 44), our study demonstrates that low CC values are correlated with mortality after discharge. In particular, after correcting for diagnosis of sarcopenia, functional status and comorbidities, high CC values were protective factor at 6 months. Previously, Ferdandes et al identified <34.5 cm as a cut-off point for estimating 9-year follow-up mortality in Brazilian subjects, higher than the value recommended in the literature (45). In their study of cancer patients, Sousa et al (46) found that CC measures below 34 cm for men and 33 cm for women were closely associated with mortality, the risk of death being 3 times greater in patients with a smaller CC. Recently, Santer et al reported that reduced CC at the first nutritional assessment increased by about the double the risk of death in the intensive care unit than those with normal CC in Brazilian older adults affected by COVID-19 (47). Finally, regarding the risk factors for mortality at T12, our results confirmed the protective role of preserved functional status (48) and the deleterious contribution of high comorbidity rate (49, 50). The fact that CC did not result as a predictor of mortality at T12 in maybe due to the relatively small sample size of our study. However, it should not be forgotten that in our sample, the average age of the participants was 83 years (minimum 65, maximum 98), so it is possible that within a year some patients will undergo a functional post-hospitalization recovery. It is therefore likely that CC predicts mortality more easily in the short term than in the long term; 12 months after discharge, other factors may intervene.

Our study has some limitations. First of all, the small sample size, which limits the statistical power of the analyses (a posteriori power analysis based on Cox regression results at T6 revealed that the sample achieved a power 0.44 at 0.05 significance level): this is a preliminary study, and future ad hoc researches are required to confirm our results. Moreover, the type of participant, i.e. hospitalized older adults, in most cases had cognitive impairment, making assessments more difficult. Age is known to significantly influence CC values, which may have had an unknow extent of impact on the study findings. We were unable to compare adequate samples of people with and without mild cognitive impairment or dementia. The Short Physical Performance Battery (SPPB) was not performed with all patients because of difficulties linked to bed rest, so an evaluation of severe sarcopenia according to EWGSOP Guidelines was not possible. On the other side, our strength was carrying out a Comprehensive Geriatric Assessment of the patients, which included an assessment of sarcopenia using BIA, and a nutritional profile, with which we could easily correlate the CC values.

From a clinical point of view, our study underlines the importance of investigating the role of CC, which could replace the use of more complex and not always manageable scores in the elderly patient. On the other hand, the detection of a CC value < 30.6 cm should suggest to the physician the adoption of an early nutritional and physical intervention in order to improve the patient's outcome, especially in the medium term.

In conclusion, calf circumference is a rapid, non-invasive, easy-to-perform, low-cost tool for assessing patients' nutritional status and skeletal muscle mass. It is the only anthropometric parameter strongly associated with the probability of mortality; its validity in predicting mortality at 3, 6 and 12 months after hospital discharge is comparable to that of MNA, one of most used tools to detect nutritional status. Finally, high CC values are protective factors when considering the risk of death at 6 months. We hope that ours can be a preliminary study to encourage further research to better define the role of this promising anthropometric index.

*Funding:* No funding was received for conducting this study. Open access funding provided by Università degli Studi di Padova within the CRUI-CARE Agreement.

*Competing interest:* The authors have no relevant financial or non-financial interests to disclose.

*Ethics approval:* The study protocol was conducted in accordance with good clinical practice guidelines and the ethical standards of the 1964 Declaration of Helsinki as revised in 2000, and was approved by the local Ethics Committee (Comitato Etico per la Sperimentazione Clinica della Provincia di Padova, protocol number 16412/AO/23).

*Consent:* The subjects taking part in the study were given a detailed explanation of the risks and benefits of participation, and all gave oral and written informed consent to publication of the data.

## Electronic supplementary material

Supplementary Figure 1. Association between calf circumference and mortality at T3, T6, T12 (smooth plots and 95% CI)

## References

[1] Evans W (1997). Symposium: Sarcopenia: Diagnosis and Mechanisms Functional and Metabolic Consequences of Sarcopenia 1. J Nutr [Internet].

[2] Zucchelli A, Manzoni F, Morandi A, Di Santo S, Rossi E, Valsecchi MG, Inzitari M, Cherubini A, Bo M, Mossello E, Marengoni A, Bellelli G, Italo-Hispanic Study Group of Delirium (2022). The association between low skeletal muscle mass and delirium: results from the nationwide multi-centre Italian Delirium Day 2017. Aging Clin Exp Res.

[3] Beaudart C, Biver E, Reginster JY, Rizzoli R, Rolland Y, Bautmans I, Petermans J, Gillain S, Buckinx F, Dardenne N, Bruyère O (2017). Validation of the SarQoL®, a specific health-related quality of life questionnaire for Sarcopenia. J Cachexia Sarcopenia Muscle.

[4] Bone AE, Hepgul N, Kon S, Maddocks M (2017). Sarcopenia and frailty in chronic respiratory disease. Chron Respir Dis.

[5] Kim S, Kim M, Lee Y, Kim B, Yoon TY, Won CW (2018). Calf Circumference as a Simple Screening Marker for Diagnosing Sarcopenia in Older Korean Adults: the Korean Frailty and Aging Cohort Study (KFACS). J Korean Med Sci.

[6] Zhang XY, Zhang XL, Zhu YX, Tao J, Zhang Z, Zhang Y, Wang YY, Ke YY, Ren CX, Xu J, Zhong Y (2019). Low Calf Circumference Predicts Nutritional Risks in Hospitalized Patients Aged More Than 80 Years. Biomed Environ Sci.

[7] Nishioka S, Yamanouchi A, Matsushita T, Nishioka E, Mori N, Taguchi S (2021). Validity of calf circumference for estimating skeletal muscle mass for Asian patients after stroke. Nutrition.

[8] Tsai AC, Lai MC, Chang TL (2012). Mid-arm and calf circumferences (MAC and CC) are better than body mass index (BMI) in predicting health status and mortality risk in institutionalized elderly Taiwanese. Arch Gerontol Geriatr.

[9] Portero-McLellan KC, Staudt C, Silva FR, Delbue Bernardi JL, Baston Frenhani P, Leandro Mehri VA (2010). The use of calf circumference measurement as an anthropometric tool to monitor nutritional status in elderly inpatients. J Nutr Health Aging.

[10] Ren C, Zhang X, Zhu Y, Xu J, Xie Y (2022). Low calf circumference can predict nutritional risk and mortality in adults with metabolic syndrome aged over 80 years. BMC Endocr Disord.

[11] Real GG, Frühauf IR, Sedrez JHK, Dall'Aqua EJF, Gonzalez MC (2018). Calf Circumference: A Marker of Muscle Mass as a Predictor of Hospital Readmission. JPEN J Parenter Enteral Nutr.

[12] Sousa-Santos AR, Afonso C, Borges N, Santos A, Padrão P, Moreira P, Amaral TF (2019). Factors associated with sarcopenia and undernutrition in older adults. Nutr Diet.

[13] González-Correa CH, Pineda-Zuluaga MC, Marulanda-Mejía F (2020). Skeletal Muscle Mass by Bioelectrical Impedance Analysis and Calf Circumference for Sarcopenia Diagnosis. J Electr Bioimpedance.

[14] Endo K, Sato T, Kakisaka K, Takikawa Y (2021). Calf and arm circumference as simple markers for screening sarcopenia in patients with chronic liver disease. Hepatol Res.

[15] Wu SE, Chen WL (2022). Calf circumference refines sarcopenia in correlating with mortality risk. Age Ageing.

[16] Li X, Lang X, Peng S, Ding L, Li S, Li Y, Yin L, Liu X (2022). Calf Circumference and All-Cause Mortality: A Systematic Review and Meta-Analysis Based on Trend Estimation Approaches. J Nutr Health Aging.

[17] Wang X, Ying Y, Pei M, Ma X, Sun Y, Wang Y, Li N (2023). Calf circumference change and all-cause mortality among community-dwelling Chinese older people. Clin Nutr.

[18] Linn BS, Linn MW, Gurel L (1968). Cumulative illness rating scale. J Am Geriatr Soc.

[19] Katz S (1983). Assessing self-maintenance: activities of daily living, mobility, and instrumental activities of daily living. J Am Geriatr Soc.

[20] Graf C (2009). The Lawton Instrumental Activities of Daily Living (IADL) Scale. Medsurg Nurs.

[21] Vellas B, Guigoz Y, Garry PJ, Nourhashemi F, Bennahum D, Lauque S, Albarede JL (1999). The Mini Nutritional Assessment (MNA) and its use in grading the nutritional state of elderly patients. Nutrition.

[22] Folstein MF, Folstein SE, McHugh PR (1975). «Mini-mental state». A practical method for grading the cognitive state of patients for the clinician. J Psychiatr Res.

[23] Chumlea WC, Roche AF, Steinbaugh ML (1985). Estimating stature from knee height for persons 60 to 90 years of age. J Am Geriatr Soc.

[24] World Health Organisation (WHO) WHO ∣ Waist Circumference and Waist-Hip Ratio. Report of a WHO Expert Consultation. Geneva, 8–11 December 2008. 2008;(December):8-11. http://www.who.int.

[25] Haase J (1946). Nutrition in dentistry. Dent Stud.

[26] Lukaski HC, Johnson PE, Bolonchuk WW, Lykken GI (1985). Assessment of fat-free mass using bioelectrical impedance measurements of the human body. Am J Clin Nutr.

[27] Kyle UG, Genton L, Hans D, Pichard C (2003). Validation of a bioelectrical impedance analysis equation to predict appendicular skeletal muscle mass (ASMM). Clin Nutr.

[28] Kyle UG, Genton L, Karsegard L, Slosman DO, Pichard C (2001). Single prediction equation for bioelectrical impedance analysis in adults aged 20–94 years. Nutrition.

[29] Cruz-Jentoft AJ, Bahat G, Bauer J, Boirie Y, Bruyère O, Cederholm T, Cooper C, Landi F, Rolland Y, Sayer AA, Schneider SM, Sieber CC, Topinkova E, Vandewoude M, Visser M, Zamboni M, Writing Group for the European Working Group on Sarcopenia in Older People 2 EWGSOP2,the Extended Group for EWGSOP2 (2019). Sarcopenia: revised European consensus on definition and diagnosis. Age Ageing.

[30] Cruz-Jentoft AJ, Landi F, Topinková E, Michel JP (2010). Understanding sarcopenia as a geriatric syndrome. Curr Opin Clin Nutr Metab Care.

[31] Senior HE, Henwood TR, Beller EM, Mitchell GK, Keogh JW (2015). Prevalence and risk factors of sarcopenia among adults living in nursing homes. Maturitas.

[32] Bianchi L, Abete P, Bellelli G, Bo M, Cherubini A, Corica F, Di Bari M, Maggio M, Manca GM, Rizzo MR, Rossi AP, Landi F, Volpato S, GLISTEN Group Investigators (2017). Prevalence and Clinical Correlates of Sarcopenia, Identified According to the EWGSOP Definition and Diagnostic Algorithm, in Hospitalized Older People: The GLISTEN Study. J Gerontol A Biol Sci Med Sci.

[33] Gong G, Wan W, Zhang X, Liu Y, Liu X, Yin J (2019). Correlation between the Charlson comorbidity index and skeletal muscle mass/physical performance in hospitalized older people potentially suffering from sarcopenia. BMC Geriatr.

[34] Tosato M, Marzetti E, Cesari M, Savera G, Miller RR, Bernabei R, Landi F, Calvani R (2017). Measurement of muscle mass in sarcopenia: from imaging to biochemical markers. Aging Clin Exp Res.

[35] Landi F, Calvani R, Coelho HJ, Ciciarello F, Galluzzo V, Zazzara B, Martone AM, Picca A, Marzetti E, Tosato M (2022). Estimated appendicular skeletal muscle mass using calf circumference and mortality: Results from the aging and longevity study in the Sirente geographic area (ilSIRENTE study). Exp Gerontol.

[36] de Onis M, Habicht JP (1996). Anthropometric reference data for international use: recommendations from a World Health Organization Expert Committee. Am J Clin Nutr.

[37] Kawakami R, Murakami H, Sanada K, Tanaka N, Sawada SS, Tabata I, Higuchi M, Miyachi M (2015). Calf circumference as a surrogate marker of muscle mass for diagnosing sarcopenia in Japanese men and women. Geriatr Gerontol Int.

[38] Rose Berlin Piodena-Aportadera M, Lau S, Chew J, Lim JP, Ismail NH, Ding YY, Lim WS (2022). Calf Circumference Measurement Protocols for Sarcopenia Screening: Differences in Agreement, Convergent Validity and Diagnostic Performance. Ann Geriatr Med Res.

[39] Srinivasaraghavan N, Venketeswaran MV, Balakrishnan K, Ramasamy T, Ramakrishnan A, Agarwal A, Krishnamurthy A (2022). Comparison of nutrition screening tools and calf circumference in estimating the preoperative prevalence of malnutrition among patients with aerodigestive tract cancers-a prospective observational cohort study. Support Care Cancer.

[40] Beretta MV, Rodrigues TDC, Steemburgo T (2023 May 29). Validity of the Global Leadership Initiative on Malnutrition criteria using calf circumference in the prediction of in-hospital mortality in older surgical patients: A secondary analysis of a cohort study. JPEN J Parenter Enteral Nutr.

[41] Luchsinger JA, Lee WN, Carrasquillo O, Rabinowitz D, Shea S (2003). Body mass index and hospitalization in the elderly. J Am Geriatr Soc.

[42] Selvaraj K, Jayalakshmy R, Yousuf A, Singh AK, Ramaswamy G, Palanivel C (2017). Can mid-upper arm circumference and calf circumference be the proxy measures to detect undernutrition among elderly? Findings of a community-based survey in rural Puducherry, India. J Family Med Prim Care.

[43] Zhang XM, Wu X, Ma Y, Zhu C, Cao J, Liu G, Li FF, Cheng ASK (2021). Comparing the Performance of Calf Circumference, Albumin, and BMI for Predicting Mortality in Immobile Patients. Risk Manag Healthc Policy.

[44] Wei J, Jiao J, Chen CL, Tao WY, Ying YJ, Zhang WW, Wu XJ, Zhang XM (2022). The association between low calf circumference and mortality: a systematic review and meta-analysis. Eur Geriatr Med.

[45] Fernandes DPS, Juvanhol LL, Lozano M, Ribeiro AQ (2022). Calf circumference is an independent predictor of mortality in older adults: An approach with generalized additive models. Nutr Clin Pract.

[46] Sousa IM, Bielemann RM, Gonzalez MC, da Rocha IMG, Barbalho ER, de Carvalho ALM, Dantas MAM, de Medeiros GOC, Silva FM, Fayh APT (2020). Low calf circumference is an independent predictor of mortality in cancer patients: A prospective cohort study. Nutrition.

[47] Santer D, Schneider N, de Carvalho YSS, de Souza Bortolini RV, Silva FM, Franken DL, da Silva Fink J (2023). The association between reduced calf and mid-arm circumferences and ICU mortality in critically ill COVID-19 patients. Clin Nutr ESPEN.

[48] Ceolin C, Bano G, Biz C, Dianin M, Bedogni M, Guarnaccia A, Berizzi A, Ruggieri P, Coin A, Sergi G (2023). Functional autonomy and 12-month mortality in older adults with proximal femoral fractures in an orthogeriatric setting: risk factors and gender differences. Aging Clin Exp Res.

[49] Formiga F, Moreno-Gonzalez R, Chivite D, Franco J, Montero A, Corbella X (2018). High comorbidity, measured by the Charlson Comorbidity Index, associates with higher 1-year mortality risks in elderly patients experiencing a first acute heart failure hospitalization. Aging Clin Exp Res.

[50] Canoui-Poitrine F, Segaux L, Benderra MA, About F, Tournigand C, Laurent M, Caillet P, Audureau E, Ferrat E, Lagrange JL, Paillaud E, Bastuji-Garin S, On Behalf Of The Elcapa Study Group (2022). The Prognostic Value of Eight Comorbidity Indices in Older Patients with Cancer: The ELCAPA Cohort Study. Cancers (Basel).

