# Supplementary material for: New Perspectives in the Association between Anthropometry and Mortality: The Role of Calf Circumference
Source: J Frailty Aging. 2024 Jan 24;13(2):108–15. doi: 10.14283/jfa.2024.4 (PMC12275705; doi:10.14283/jfa.2024.4)
Supplement: Supplementary file 1 — Supplementary Figure 1. Association between calf circumference and mortality at T3, T6, T12 (smooth plots and 95% CI) [file mmc1.docx]

**Supplementary Figure 1. Association between calf circumference and mortality at T3, T6, T12 (smooth plots and 95% CI)**


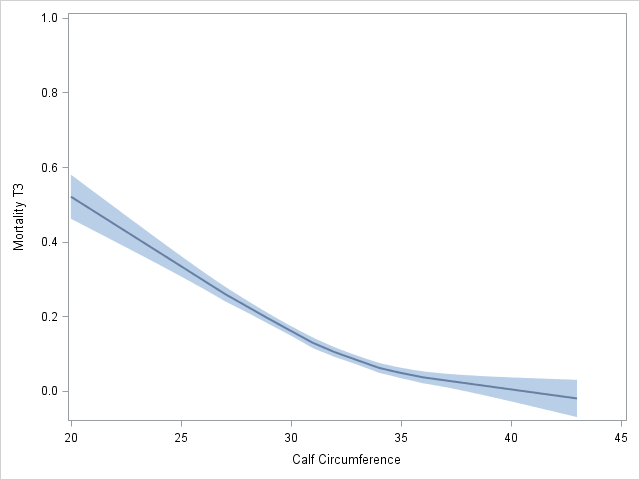


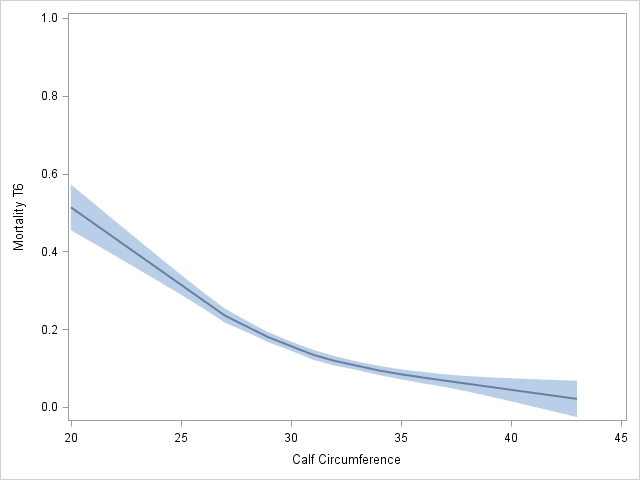


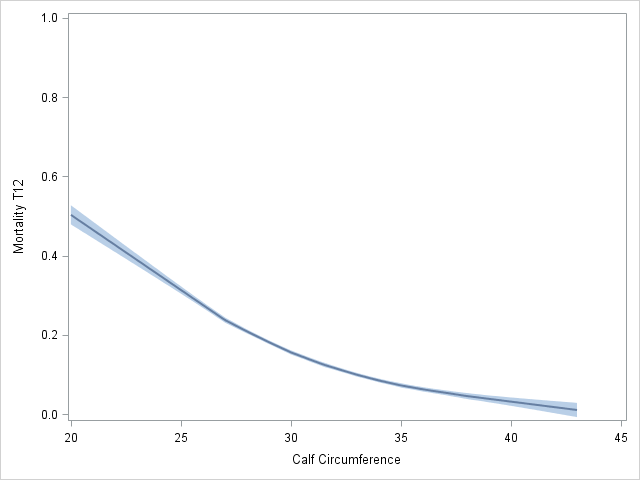


*Notes*: T3: 3 months after discharge; T6: 6 months after discharge; T12: 12 months after discharge. Calf circumference is measured in centimeters (cm).
